# Participation of the elderly, women, and minorities in pivotal trials supporting 2011–2013 U.S. Food and Drug Administration approvals

**DOI:** 10.1186/s13063-016-1322-4

**Published:** 2016-04-14

**Authors:** Nicholas S. Downing, Nilay D. Shah, Joseph H. Neiman, Jenerius A. Aminawung, Harlan M. Krumholz, Joseph S. Ross

**Affiliations:** Center for Outcomes Research and Evaluation, Yale–New Haven Hospital, New Haven, CT USA; Division of Health Care Policy and Research, Mayo Clinic, Rochester, MN USA; University of Nevada School of Medicine, Reno, NV USA; Section of General Internal Medicine and the Robert Wood Johnson Foundation Clinical Scholars Program, Department of Internal Medicine, Yale University School of Medicine, New Haven, CT USA; Section of Cardiovascular Medicine and the Robert Wood Johnson Foundation Clinical Scholars Program, Department of Internal Medicine, Yale University School of Medicine, New Haven, CT USA; Department of Health Policy and Management, Yale University School of Public Health, New Haven, CT USA; Section of General Internal Medicine and the Robert Wood Johnson Foundation Clinical Scholars Program, Department of Internal Medicine, Yale University School of Medicine, New Haven, CT USA

## Abstract

**Background:**

Pivotal trials, the clinical studies that inform U.S. Food and Drug Administration (FDA) approval decisions, provide the foundational evidence supporting the safety and efficacy of novel therapeutics. We determined the representation of the elderly, women, and patients from racial and ethnic minorities in pivotal trials and whether the FDA is making subgroup efficacy analyses among these subpopulations available to the public.

**Methods:**

We conducted a cross-sectional study of novel therapeutics approved by the FDA between 2011 and 2013. Using publicly available FDA documents, we collected information on the demographic characteristics of pivotal trial participants (age ≥65 years, sex [male, female], race [white, black, Asian, other], and ethnicity [Hispanic, non-Hispanic]) and determined the availability of subgroup analyses by age, sex, race, and ethnicity.

**Results:**

We identified 86 novel therapeutic that were approved by the FDA between 2011 and 2013 for 92 indications on the basis of 206 pivotal trials. The median age of pivotal trial patients was 53.1 years (interquartile range 40.6–60.6), and the mean proportion of patients ≥65 years of age was 28.9 % (95 % CI 23.5–34.4 %). Similar proportions of pivotal trial participants were male (mean 50.3 %, 95 % CI 45.3–55.2 %) and female (mean 49.7 %, 95 % CI 44.7–54.7 %). Most participants were white (mean 79.2 %, 95 % CI 75.9–82.6 %), while the mean proportion of black patients was 7.4 % (95 % CI 5.5–9.3 %), that of Asian patients was 7.4 % (95 % CI 5.2–9.7 %), and that of patients of other races was 5.9 % (95 % CI 4.4–7.5 %). Information about ethnicity was available for only 59.8 % of indications, and where such data were available, the mean proportion of Hispanic participants was 13.3 % (95 % CI 10.3–16.3 %). FDA reviewers performed and made available subgroup efficacy analyses by age, sex, and race for at least one of the pivotal trials used as the basis of approval for over 80 % of indications.

**Conclusions:**

Although women are equally represented in pivotal trials supporting recent novel therapeutic approvals by the FDA, elderly patients and those from racial and ethnic minorities are underrepresented. FDA reviewers generally perform subgroup efficacy analyses by age, sex, and race and make these subgroup analyses available to the public.

## Background

Clinical trials provide the foundational evidence that supports U.S. Food and Drug Administration (FDA) approval and informs physicians and patients as they make decisions about the use of new therapies. For this evidence to inform both regulators and clinical practice, it is critical that the patients participating in these trials, wherever they are conducted, reflect the population of patients expected to use the novel therapeutic agent. This is particularly important for pivotal clinical trials, the late-stage clinical trials that are intended to demonstrate the safety and efficacy of novel therapeutics and upon which the FDA bases its approval decisions [1]. However, several groups of individuals, including the elderly, women, and persons from racial and ethnic minority groups, have historically been underrepresented in clinical research, limiting the ability of many studies to inform decisions about their care [2–8].

Over the years, the FDA has taken several measures aimed at improving and ensuring the representativeness of patients participating in pivotal clinical trials. First, in 1998, the FDA issued the “demographic rule,” requiring drug makers to report the age, sex, and race of clinical trial participants in their annual reports to the agency [9]. While the demographic rule ensured that this information was available to the FDA, it did not mandate its availability to the public. However, the 2012 FDA Safety and Innovation Act (FDASIA) urged the FDA to address this issue, and, in August 2013, the agency released a report describing the demographic characteristics of participants in trials of drugs and devices approved in 2011, as well as the availability of subgroup analyses for key age, sex, and race subgroups [10]. More recently, the FDA published an expanded investigation of the representativeness of clinical trials supporting drugs approved between 2010 and 2012 [11].

In this article, we describe the results of our research to characterize the demographic characteristics of patients participating in pivotal clinical trials for all novel therapeutics approved by the FDA between 2011 and 2013 and to determine the availability of subgroup efficacy analyses using publicly available data sources. Our research was similarly focused on participation in these trials by members of historically underrepresented populations: the elderly (defined as patients aged 65 years or older), women, persons of nonwhite race, and Hispanics. Although our study was initiated before the publication of the FDA’s initial report prompted by the FDASIA and subsequent research study [10, 11], there are three specific reasons that support the current dissemination of our work: (1) to validate the FDA’s findings of the age, sex, and race of clinical trial participants, exclusively using publicly available data sources, since the FDA’s study made use of internal documents submitted to the agency that are not available to the public; (2) to confirm the FDA’s findings among 2013 drug approvals, since the FDA’s study focused on 2010 through 2012 approvals; and (3) to provide a broader investigation of the issue by examining trial participation by ethnicity, since the FDA study focused only on age, sex, and race.

## Methods

### Study sample

Using the Drugs@FDA database, a publicly available index of FDA regulatory actions, we identified all novel therapeutics (i.e., new molecular entities—drugs—and novel biologics) that were approved by the FDA between 1 January 2011 and 31 December 2013 [12]. Generic drugs, reformulations, combinations of nonnovel therapeutic agents, and nontherapeutic agents (i.e., diagnostic agents) were excluded. For each of the novel therapeutics included in our sample, we determined the indications for which it was first approved for use by reviewing the FDA’s approval letter and the initial drug label. All indications were classified into one of eight therapeutic areas (cancer, cardiovascular [including diabetes mellitus and hyperlipidemia], infectious disease, autoimmune and musculoskeletal disease, neurology, dermatology, psychiatry, and other). Next, we determined whether these novel therapeutics had been granted orphan status, indicating that they are primarily intended for the treatment of a rare disease. For small molecules, orphan status was determined using the Drugs@FDA database [12]; for biologics, we searched the Orphan Drug Product Designation Database [13].

### Pivotal trials

The FDA makes a series of documents available in the Drugs@FDA database that correspond to the key components of the agency’s review process, explain the basis of its approval decision, and summarize notable information presented in sponsors’ applications. The “medical review” discusses the safety and efficacy of novel therapeutics, describing the pivotal trials in detail. For each of the novel therapeutics included in our sample, we manually searched the corresponding medical review documents to identify the pivotal trials that supported their approval and recorded the intention-to-treat (ITT) population (i.e., all participants who received at least one dose of the study drug or its comparator). If the medical review did not clearly identify the pivotal trials used as the basis of approval, we reviewed other FDA documents to identify such trials, such as the summary review and office director memos, and if necessary manually classified trials as pivotal following an established approach [1].

### Demographic characteristics of pivotal trial participants

Information describing the age, sex, race, and ethnicity of the participants in each of the identified pivotal trials was abstracted from the FDA’s medical review documents. In circumstances when the medical review did not contain all of this information, the agency’s statistical reviews were searched. To characterize the ages of pivotal trial participants, we recorded the mean as a summary statistic; if the mean was unavailable, the median was used. When FDA documents provided such statistics for individual trial arms, but not in the aggregate, we used a weighted-average approach to estimate the corresponding statistic for the entire study population. In addition, we determined the number of elderly clinical trial participants, defined as those aged 65 years or older. Next, we recorded the number of male and female participants in each study. To characterize clinical trial participants’ race and ethnicity, we counted the number of participants in four racial subgroups (white, black, Asian, and other) and two ethnic subgroups (Hispanic and non-Hispanic). Two reviewers (JHN and JAA) independently abstracted this information from FDA documents. A second reviewer (NSD) validated the initial abstraction by reabstracting all data, and all discrepancies were resolved through discussion with the senior investigator (JSR).

### Availability of subgroup efficacy analyses

To determine whether FDA reviewers documented the efficacy of novel therapeutics for age, sex, and race as well as ethnic subgroups, we searched the medical and statistical reviews to identify the presence of subgroup analyses for each pivotal trial. Analyses of efficacy involving patients of any two age groups (e.g., <40 years vs. ≥40 years; <65 years vs. ≥65 years) were considered to represent a subgroup analysis by age. We considered an acknowledgement of insufficient sample size in certain subgroups to represent subgroup analysis because the agency made an attempt to produce this information.

### Statistical analysis

For each pivotal trial, we computed the proportions of patients in various age (≥65 years), sex (male, female), race (white, black, Asian, other), and ethnicity (Hispanic, non-Hispanic) subgroups. Next, to reflect the overall demographic composition of the clinical development program for each novel therapeutic, we aggregated these data, which describe the characteristics of patients participating in individual pivotal trials, to the indication level. To do this, we weighted the contribution from each pivotal trial according to its ITT population. In cases where certain data fields were available for some but not all of the pivotal trials supporting an indication, we aggregated only data from trials for which the field was available; for fields that were not available for any pivotal trials within an indication, we noted that the data were missing.

After aggregating this information to the indication level, we characterized the demographic composition of pivotal trial patients using descriptive statistics, reporting the median and interquartile range (IQR) for age and computing the mean proportion of patients in the various age, sex, race, and ethnicity subgroups with the corresponding 95 % CIs. In addition, we performed a stratified analysis by therapeutic area and orphan status. To contextualize our results, we also computed summary statistics for the corresponding demographics of the U.S. population using data derived from 2010 U.S. Census [14]. Last, we counted the number of indications for which analyses of efficacy in age, sex, race, and ethnicity subgroups were documented in the FDA’s review of at least one of the associated pivotal trials.

Since the epidemiology of a disease may preclude recruitment of pivotal trial participants of differing ages, sexes, races, and ethnicities, we repeated all analyses after excluding those therapeutics indicated for diseases that are uncommon among historically underrepresented populations, including the elderly, females, and racial and ethnic minorities. For instance, a pivotal trial for a novel therapeutic used to treat prostate cancer would not be expected to recruit women. To identify such therapeutics, two investigators (NSD and JSR) independently reviewed the indications for which each novel therapeutic was initially approved and, based on their clinical experience, excluded those where the disease prevalence in the four historically underrepresented populations was approximately less than half the disease prevalence among the “majority” populations. As another example, ivacaftor, a drug for the treatment of cystic fibrosis, a disease that affects primarily young white individuals, was excluded from analyses examining age, race, and ethnicity subgroups, because it is unrealistic to expect that pivotal trials involving this drug would include elderly, nonwhite, or Hispanic patients. (For a complete list of therapeutic subgroup population exclusions, see Tables 5, 6 and 7 in Appendix). All data were collected and analyzed using Microsoft Excel software (Microsoft, Redmond, WA, USA). Statistical analysis was performed using JMP software (SAS Institute, Cary, NC, USA).

## Results

We identified 89 novel therapeutics that the FDA approved between 2011 and 2013 for use in 97 indications. (For details of sample construction, see Fig. 2 in the Appendix) After removing therapeutics and indications that were approved in the absence of a pivotal trial, our final sample consisted of 86 therapeutics approved for use in 92 indications. Of these 86 therapeutics, 27 (31.4 %) were approved in 2011, 35 (40.7 %) in 2012, and 24 (27.9 %) in 2013 (Table 1). Drugs approved for indications in cancer (30.4 %), cardiovascular disease (12.0 %), and infectious disease (9.8 %) accounted for over half of approvals, while 30 (32.6 %) indications received orphan status.

**Table 1:** Novel therapeutic agents and Associated Indications Approved by the U.S. Food and Drug Administration between 2011 and 2013

	Number (%)
Novel therapeutics, *n* = 86	
Approval year	
2011	27 (31.4 %)
2012	35 (40.7 %)
2013	24 (27.9 %)
Number of approved indications	
One	80 (93.0 %)
Two	5 (5.8 %)
Three	1 (1.2 %)
Associated indications, *n* = 92	
Therapeutic area	
Cancer	28 (30.4 %)
Cardiovascular disease, diabetes mellitus, hyperlipidemia	11 (12.0 %)
Infectious disease	9 (9.8 %)
Autoimmune and musculoskeletal	4 (4.3 %)
Neurology	7 (7.6 %)
Dermatology	5 (5.4 %)
Psychiatry	2 (2.2 %)
Other	26 (28.3 %)
Orphan status	
Yes	30 (32.6 %)
No	62 (67.4 %)
Number of pivotal trials	
One	41 (41.6 %)
Two	28 (30.4 %)
Three	11 (12.0 %)
Four	12 (13.0 %)

The 92 indications were approved on the basis of 206 pivotal trials that included a total of 174,855 patients. The number of pivotal trials supporting each approval varied: 41 (44.6 %) indications were approved on the basis of a single pivotal trial, 28 (30.4 %) on the basis of two pivotal trials, and 23 (25.0 %) on the basis of three or more pivotal trials. When aggregated to the indication level, the mean number of patients enrolled in the pivotal trials supporting each approved indication was 1900 (standard deviation 3156).

### Demographic characteristics of pivotal trial participants

**Fig. 1:**
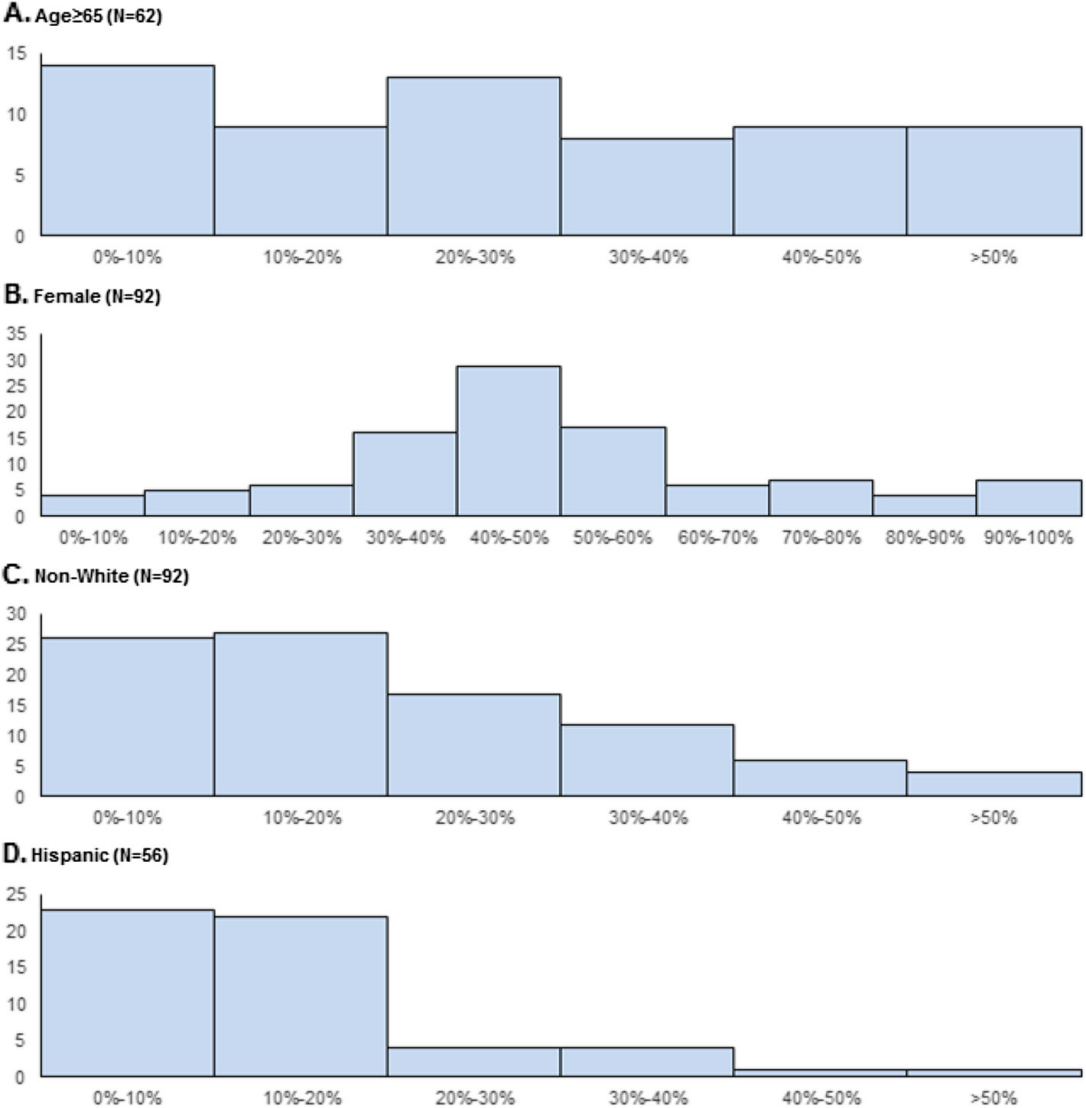
Number of pivotal trials that supported the approval of novel therapeutics approved between 2011 and 2013, by the proportion of participating elderly, female, nonwhite and Hispanic patients

**Fig. 2:**
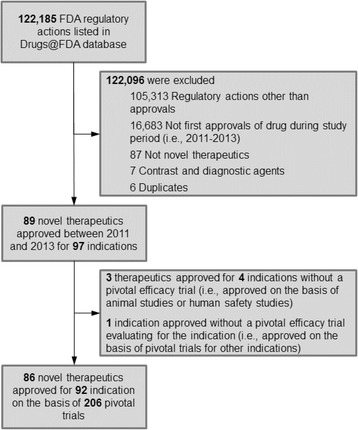
Sample construction of novel therapeutic agents approved by the U.S. Food and Drug Administration (FDA) between 2011 and 2013, including associated indications and identified pivotal trials

At the indication level, the median age of pivotal trial patients was 53.1 years (IQR 40.6–60.6) (Table 2). Subgroup categorization of participants’ age was available for at least one trial supporting 61 (66.3 %) of 92 indications, and among these indications, the mean proportion of patients aged ≥65 years was 28.9 % (95 % CI 23.5–34.4 %). For 9 indications (14.5 %), more than half of the patients were aged 65 years or older (Fig. 1). Of the 92 indications, 22 (23.9 %) are used to treat diseases that are uncommon in the elderly. When we limited our analyses to indications for which the inclusion of the elderly should be expected, we found fairly consistent results (Table 2).

**Table 2:** Characteristics of participants in pivotal trial aggregated at the indication level providing the basis of U.S. Food and Drug Administration approval of novel therapeutic agents between 2011 and 2013

	All indications	Indications that should involve underrepresented patient groups^a^	U.S. population
Age			
Median^b^ (IQR)	53.1 (40.6–60.6)	56.4 (50.5–62.5)	37.2 (*n/a*)
Aged ≥65 years^c^, proportion of patients (95 % CI)	28.9 % (23.5–34.4 %)	32.6 % (26.9–38.4 %)	13.0 %
Sex, proportion of patients (95 % CI)			
Male	50.3 % (45.3–55.2 %)	46.4 % (41.8–50.9 %)	49.2 %
Female	49.7 % (44.7–54.7 %)	53.6 % (49.0–58.2 %)	50.8 %
Race, proportion of patients (95 % CI)			
White	79.2 % (75.9–82.6 %)	77.7 % (74.3–81.2 %)	72.5 %
Black	7.4 % (5.5–9.3 %)	8.0 % (6.0–10.0 %)	13.2 %
Asian	7.4 % (5.2–9.7 %)	7.9 % (5.4–10.3 %)	5.4 %
Other^d^	5.9 % (4.4–7.5 %)	6.4 % (4.7–8.0 %)	8.8 %
Ethnicity, proportion of patients^e^ (95 % CI)			
Hispanic	13.3 % (10.3–16.3 %)	n/a	16.3 %
Non-Hispanic^d^	86.7 % (83.7–89.7 %)	n/a	83.7 %

*n/a* not applicable

^a^Includes only those indications for which the prevalence of the disease is evenly spread across patients of all ages, sexes, or races. Indications for which the prevalence in three key demographic subgroups (age ≥65 years, female sex, nonwhite race) is less than half that of the prevalence among other patients were excluded.

^b^Mean age was unavailable for 28 trials. In these cases, we assumed that the median age, which was universally reported for these trials, approximated the mean.

^c^Includes patients in subgroups defined as >65 as well as ≥65 years old. Age breakdown was not available for 31 (33.7 %) of 92 in overall analysis and 19 (27.1 %) of 70 in the sensitivity analysis.

^d^Includes patients whose race/ethnicity was not explicitly defined

^e^Ethnicity information was not available for 37 (40.2 %) of 92 indications in overall analysis. Sensitivity analysis by ethnicity was not performed.

Information about the sex of clinical trial participants was universally available, and equal proportions of clinical trial participants were male (50.3 %, 95 % CI 45.3–55.2 %) and female (49.7 %, 95 % CI 44.7–54.7 %), although there was variation: in 12.0 % of indications, over 80 % of patients were female, and in 9.8 % of indications, over 80 % were male. Of the 92 indications, 14 (15.2 %) are used to treat diseases that are uncommon in women. When we limited our analyses to indications for which the inclusion of women should be expected, we found fairly consistent results (Table 2).

Approximately 80 % of clinical trial participants were white (79.2 %, 95 % CI 75.9–82.6 %), while 7.4 % (95 % CI 5.5–9.3 %) were black, 7.4 % (95 % CI 5.2–9.7 %) were Asian, and 5.9 % (95 % CI 4.4–7.5 %) were of other races. When compared with the overall U.S. population, more patients participating in clinical trials included in our study were white (79.2 % vs. 75.2 %) and Asian (7.4 % vs. 5.4 %), while fewer patients were black (7.4 % vs. 13.2 %). Nonwhite patients comprised 30 % or more of the clinical trial participants for almost one-fourth of indications (22 of 92, or 23.9 %). In contrast to information about the age, sex, and race of clinical trial participants, which was almost always available, information about the ethnicity of clinical trial participants was available for only 55 (59.8 %) of 92 indications. Among indications where these data were reported, 13.3 % (95 % CI 10.3–16.3 %) were Hispanic, which is less than the 16.2 % of the overall U.S. population. Hispanic participants comprised 20 % or less of participants in clinical trials supporting the approval of 81.8 % (45 of 55) of indications where ethnicity information was reported. Of the 92 indications included in this analysis, 14 (15.2 %) are used to treat diseases that are uncommon in racial minorities. When we limited our analyses to indications for which the inclusion of patients from racial minorities should be expected, we found fairly consistent results (Table 2).

### Pivotal trial participants by therapeutic area

There was significant variation in the characteristics of patients participating in clinical trials according to therapeutic area (Table 3). Pivotal trials involving therapies for cancer tended to involve higher proportions of elderly patients than those for other indications (36.6 % [95 % CI 27.6–45.6 %] vs. 23.9 % [95 % CI 17.3–30.6 %]), higher proportions of white patients (82.6 % [95 % CI 76.4–88.8 %] vs. 77.8 % [95 % CI 73.7–81.8 %]), and lower proportions of Hispanic patients (3.3 % [95 % CI 0.5–6.0 %] vs. 10.0 % [95 % CI 7.1–12.9 %]). Male patients were more common in pivotal trials of therapies for infectious diseases than for other indications (66.2 % [95 % CI 55.5–76.8 %] vs. 48.6 % [95 % CI 43.2–53.9 %]), and a far higher proportion of black patients also participated in such trials (16.7 % [95 % CI 8.7–24.7 %] vs. 9.5 % [95 % CI 4.5–8.3 %]).

**Table 3:** Characteristics of participants in pivotal trials at the indication level providing the basis of U.S. Food and Drug Administration approval of novel therapeutic agents between 2011 and 2013, stratified by indication-level characteristics

	Therapeutic area								Orphan status	
	Cancer (*n* = 28)	Cardiovascular disease, diabetes mellitus, hyperlipidemia (*n* = 11)	Infectious disease (*n* = 9)	Autoimmune and musculoskeletal (*n* = 4)	Neurology (*n* = 7)	Dermatology (*n* = 5)	Psychiatry (*n* = 2)	Other (*n* = 26)	Yes	No
Trial size, mean (SD)										
ITT population	490 (360)	6080 (6708)	1368 (623)	1829 (1698)	1181 (789)	415 (106)	2301 (2013)	2295 (2560)	323 (249)	2664 (3607)
Age										
Median, years (IQR)	57.6 (52.2–62.4)	57.1 (50.7–62.2)	47.1 (37.0-50.4)	43.5 (37.6–52.0)	37.7 (34.8–49.9)	40.6 (24.8–65.1)	44.3 (40.9–47.7)	56.2 (44.4–62.5)	51.5 (39.5–59.5)	54.6 (43.1–61.6)
Aged ≥65 years, proportion of patients (95 % CI)	36.6 % (27.6–45.6 %)	35.1 % (23.1–47.0 %)	12.0 % (0.0–38.1 %)	11.4 % (0.0–32.8 %)	1.5 % (0.0–100.0 %)	35.3 % (0.0–100.0 %)	1.0 % (n/a)	25.4 % (14.5–36.4 %)	30.0 % (21.7–38.2 %)	28.3 % (20.8–35.7 %)
Sex, mean proportion of patients (95 % CI)										
Male	56.2 % (46.9–65.8 %)	46.2 % (35.8–56.8 %)	66.2 % (55.5–76.8 %)	31.6 % (0.0–75.3 %)	43.3 % (32.3–54.3 %)	64.9 % (27.0–100.0 %)	37.5 % (0.0–77.8 %)	53.6 % (32.5–53.6 %)	51.3 % (46.2–56.3 %)	49.8 % (42.8–57.9 %)
Female	43.8 % (34.2–53.4 %)	53.8 % (43.3–64.2 %)	33.9 % (23.2–44.5 %)	68.4 % (24.8–100.0 %)	56.8 % (45.8–67.7 %)	35.1 % (0.0–73.0 %)	62.5 % (22.2–100.0 %)	56.9 % (46.4–67.5 %)	48.8 % (43.7–53.8 %)	50.2 % (43.1–57.2 %)
Race, mean proportion of patients (95 % CI)										
White	82.6 % (76.4–88.8 %)	71.2 % (63.6–78.8 %)	71.9 % (52.8–91.1 %)	68.4 % (38.8–98.0 %)	81.7 % (70.6–92.8 %)	74.5 % (45.3–100.0 %)	83.3 % (59.1–100.0 %)	83.1 % (78.2–88.1 %)	78.0 % (70.2–85.8 %)	79.8 % (76.4– 83.2 %)
Black	4.1 % (2.1–6.2 %)	4.6 % (0.3–8.3 %)	16.7 % (8.8–24.7 %)	5.8 % (0.0–12.9 %)	3.2 % (0.0–6.4 %)	15.0 % (0.0–40.5 %)	10.9 % (0.0–49.5 %)	8.6 % (4.6–12.6 %)	5.4 % (2.8–8.2 %)	8.3 % (5.8–10.9 %)
Asian	9.0 % (3.0-15.0 %)	17.5 % (9.6–25.3 %)	4.3 % (1.2–7.4 %)	12.4 % (0.0–28.1 %)	9.3 % (0.0–18.4 %)	0.4 % (0.0–1.0 %)	4.3 % (0.0–25.8 %)	2.9 % (1.4–4.4 %)	10.6 % (4.9–16.4 %)	5.9 % (3.8–7.9 %)
Other^a^	4.2 % (2.8–5.7 %)	7.0 % (2.8–11.2 %)	7.0 % (0.0–17.5 %)	13.5 % (2.0–24.9 %)	5.7 % (0.1–10.4 %)	10.1 % (0.0–28.9 %)	1.8 % (0.0–7.8 %)	5.4 % (3.0–7.8 %)	5.9 % (2.9–8.9 %)	6.0 % (4.2–7.7 %)
Ethnicity, mean proportion of patients (95 % CI)										
Hispanic	7.6 % (1.7–13.5 %)	12.6 % (7.4–17.7 %)	15.3 % (11.6–18.9 %)	35.2 % (n/a)	17.1 % (0.0–43.8 %)	42.3 % (15.4–69.2 %)	6.8 % (n/a)	10.7 % (7.1–14.2 %)	10.9 % (4.1–17.6 %)	14.1 % (10.6–17.5 %)
Non-Hispanic^a^	92.4 % (86.5–98.3 %)	87.4 % (82.3–92.6 %)	84.7 % (81.0–88.4 %)	64.7 % (n/a)	82.7 % (56.6–100.0 %)	57.5 % (30.7–84.3 %)	93.1 % (n/a)	89.3 % (85.8–92.9 %)	89.1 % (82.4–95.9 %)	85.9 % (82.5–89.4 %)

*n/a* not applicable

*Note*: All indications included

^a^Includes patients whose race/ethnicity was not explicitly defined

### Pivotal trial participants by orphan status

The age and sex of participants in clinical trials involving therapies receiving orphan status tended to be similar to those that did not receive orphan status. Although there were comparable proportions of white patients participating in clinical trials of drugs receiving orphan status, the proportion of black patients was lower (5.4 % [95 % CI 2.8–8.2 %] vs. 8.3 % [95 % CI 5.8–10.9 %]), while the proportion of Asian patients was higher (10.6 % [95 % CI 4.9–16.4 %] vs. 5.9 % [95 % CI 3.8–7.9 %]). Hispanic patients tended to be underrepresented in clinical trials involving therapies receiving orphan status. The mean proportions of Hispanic patients were 10.9 % (95 % CI 4.1–17.6 %) for therapies with orphan status compared with 14.1 % (95 % CI 10.6–17.5 %) for therapies without orphan status.

### Availability of subgroup efficacy analyses

FDA reviewers generally performed subgroup efficacy analyses by age, sex, and race for at least one of the pivotal trials used as the basis of approval for most indications included in our sample (Table 4). Specifically, subgroup analyses by age were performed for 85 (92.4 %) of 92 indications, by sex for 80 (87.0 %) of 92 indications, and by race 74 (80.4 %) of 92 indications. In contrast, subgroup analyses by ethnicity were performed in 21 (22.8 %) of 92 indications.

**Table 4:** Availability of at least one subgroup analysis for each indication associated with novel therapeutic agents approved by the U.S. Food and Drug Administration between 2011 and 2013

Subgroup analysis reported in medical or statistical review	All indications, *n* (%)	Indications which involve patients across subgroup,^a^ *n* (%)
Age	85/92 (92.4 %)	65/70 (92.9 %)
Sex	80/92 (87.0 %)	76/84 (90.5 %)
Race	74/92 (80.4 %)	73/84 (86.9 %)
Ethnicity	21/92 (22.8 %)	n/a

^a^Includes only those indications for which the prevalence of the disease is evenly spread across patients of all ages, sexes, or races. Indications for which the prevalence in three key demographic subgroups (age ≥65 years, female sex, nonwhite race) was less than half that of the prevalence among other patients were excluded.

After removing indications involving disease processes that are uncommon in the elderly, females, and racial minorities, the rates with which the FDA reviewer performed subgroup analyses were consistent by age, sex, and race, but rose slightly.

## Discussion

Our characterization of the demographic characteristics of patients participating in pivotal trials for all novel therapeutics approved by the FDA between 2011 and 2013 demonstrates that female patients, and to a certain extent elderly patients, appear to be appropriately represented, while black and Hispanic patients remain underrepresented. Specifically, we found that elderly patients made up between one-fourth and one-third of all pivotal trial participants and that the proportion of participating male and female patients was equal. White patients accounted for almost 80 % of pivotal trial participants, a higher proportion than in the general population; in contrast, between 5 % and 10 % of patients participating in pivotal trials were black, far less than their representation in the general population, and similarly low rates of participation were observed for Hispanic patients. Last, we found that the FDA’s subgroup efficacy analyses by age, sex, and race were usually but not always provided within publicly available documents, while such analyses by ethnicity were seldom available.

Our results suggest that information describing the sex of pivotal trial participants is being made available within FDA documentation and that women are equally represented in more recently conducted pivotal clinical trials. These findings suggest that progress has been made since the early 2000s, when the participation of women in clinical research was limited [3, 4, 6–8]. For example, in our present study we found that among therapeutics approved for cardiovascular disease, almost 45 % of participants in pivotal trials were female; in contrast, women comprised only 30 % of the trials used to inform the American Heart Association’s 2007 guideline for the prevention of cardiovascular disease [15].

Similarly, it appears that more elderly patients are being recruited to participate in clinical trials than in the past; however, rates are still unlikely to approximate disease burden. For example, the proportion of elderly patients in pivotal trials involving novel therapeutics for cancer was approximately 36 %. While this represents an improvement over trials conducted between 1996 and 1998, in which elderly patients accounted for 25 % of participants [5], the age-adjusted incidence rate of cancer is 10 times higher among those aged 65 years or older [16].

In contrast, we observed that the proportion of black patients participating in pivotal trials was relatively low, below rates that would be expected given the general population and well below rates that would be expected given disparities in disease burden, as minority populations are more likely to be diagnosed with many known diseases, including cancer, heart disease, and diabetes [17]. This finding indicates that racial disparities in clinical research participation that have been noted in the 1990s and 2000s likely persist today [4, 5, 8]. The limited reporting of ethnicity information and the apparent underrepresentation of Hispanic patients may reflect the fact that only in 2005 did the FDA clarify its expectation that pivotal trial participant ethnicity be documented [18]. Nevertheless, little is known about the study of historically underrepresented minorities in clinical trials after FDA approval, suggesting that the FDA approval itself offers an important opportunity to ensure adequate representation of pivotal trial populations.

Our findings validate the FDA’s research that characterized the demographic characteristics of participants in pivotal trials of drugs approved between 2010 and 2012 [10, 11] and demonstrate that the observations made in the agency’s analyses extend to drugs approved in 2013. We found that the availability of subgroup analyses of efficacy by age, sex, and race was slightly lower in our study than the FDA’s and could be explained by the FDA’s use of nonpublic data sources. In the aggregate, our study and the FDA’s research indicate that it may be possible for the agency to do more to encourage the participation of the elderly and racial and ethnic minorities in pivotal trials. However, the inclusion of such patients in clinical trials is not enough; subgroup efficacy analyses ought to be made more easily available to the public, rather than just in the agency’s lengthy review documents. The FDA took an important step forward in August 2014 by releasing an action plan to address the issue of demographic subgroup participation and the availability of the associated data [19]. In addition, the launch of the FDA’s “Drug Trials Snapshot” website provides straightforward information about the demographic characteristics of pivotal trial participants and the results of subgroup analyses of efficacy; however, this useful tool is available for only a handful of drugs at the moment [20].

There are several important limitations of this study to consider. First, we describe the demographic characteristics of participants in pivotal trials only. While these trials represent the primary source of information characterizing the safety and efficacy of novel therapeutics, it is possible that the demographic composition of other trials, which were not considered to be as important to regulators, may be different and could potentially include higher proportions of elderly and female patients as well as those from racial and ethnic minorities. Second, our analysis was focused on the public availability of information about the demographic characteristics of patients participating in clinical trials. Since applications submitted to the FDA are not available to the public, the agency may have additional insight into the demographics of pivotal trial participants that are not described in its publicly available review documents, which were the basis of our study. Moreover, any information is made available for only therapeutics approved by the FDA; we were unable to examine the demographic characteristics of participants in pivotal trials of unapproved therapeutics. Third, we did not determine whether the FDA subgroup efficacy analysis identified differences among population subgroups, only whether the analysis was performed. Nor did we determine whether the FDA conducted subgroup safety analyses, which the FDA should consider as part of new drug evaluations as directed by the FDASIA.

Fourth, our assessments of the representativeness of patients participating in clinical trials were necessarily crude and relied on comparisons with the demographics of the overall U.S. population. Ideally, the characteristics of pivotal trial participants would be compared with the age, sex, race, and ethnicity of the population of patients affected by the disease for which each novel therapeutic was indicated; however, with the exception of cancer, such detailed epidemiological data are not systematically available. Fifth, the location of the sites where these clinical trials were conducted may influence the demographic composition of pivotal trial participants. Information on locations of clinical trial sites was not systematically available in the FDA documents, such that we were unable to characterize clinical trial participants on the basis of clinical trial site locations. While many consider drug development and evaluation to be a global enterprise, the demographics of the pivotal trial participants should be reasonably expected to reflect the U.S. patient population for whom the therapeutic is being approved for use. We cannot be certain whether our findings suggesting that more elderly patients are being recruited to participate in clinical trials than in the past, but that black and Hispanic patients remain underrepresented, are a consequence of greater trial recruitment from non-U.S. countries or other reasons. Last, in our present study, we relied on the demographic data reported in FDA documents. It is not known what methods were used to ascertain this demographic data, originally collected by each manufacturer, or whether the data collection process was standardized across manufacturers. However, the FDA does suggest that patients participating in clinical trials self-report their race and ethnicity [18].

## Conclusions

Although women are equally represented in pivotal trials supporting recent novel therapeutic approvals by the FDA, elderly patients and those from racial and ethnic minorities are underrepresented. FDA reviewers generally perform subgroup analyses by age, sex, and race and make these subgroup analyses available to the public, but not in the most accessible formats. More can be done to ensure adequate representation of pivotal trial populations to inform clinical decision-making at the time of drug approval.

## Appendix

**Table 5:** Indications excluded from selected analyses^a^ on the basis of disproportionately low disease prevalence among those aged ≥65 years

Drug	Indication
Rilpivirine hydrochloride	Human immunodeficiency Virus
Spinosad	Lice
Telaprevir	Hepatitis C
Brentuximab vedotin	Hodgkin lymphoma
Brentuximab vedotin	Large-cell lymphoma
Clobazam	Lennox-Gastaut syndrome
Boceprevir	Hepatitis C
Belimumab	SLE
Lucinactant	Respiratory distress syndrome
Taliglucerase alfa	Gaucher disease
Ivacaftor	Cystic fibrosis
Crofelemer	Patients with diarrhea and Human immunodeficiency Virus infection on antiretroviral therapy
Teriflunomide	Relapsing multiple sclerosis
Cobicistat; elvitegravir; emtricitabine; tenofovir disoproxil fumarate	Human immunodeficiency Virus
Lomitapide mesylate	Homozygous familial hypercholesterolemia
Mipomersen sodium	Homozygous familial hypercholesterolemia
Dimethyl fumarate	Relapsing remitting multiple sclerosis
Macitentan	Pulmonary arterial hypertension (WHO group 1)
Dolutegravir sodium	Human immunodeficiency Virus
Riociguat	Persistent/recurrent CTEPH (WHO group 4) after surgery or inoperable CTEPH to improve exercise capacity and WHO functional class
Riociguat	Pulmonary arterial hypertension (WHO group 1) to improve exercise capacity, WHO class, delay functional worsening
Simeprevir sodium	Hepatitis C

*CTEPH* chronic thromboembolic pulmonary hypertension, *SLE* systemic lupus erythematosus, *WHO* World Health Organization

^a^As described in detail within the main text

**Table 6:** Indications excluded from selected analyses^a^ on the basis of disproportionately low disease prevalence among females

Drug	Indication
Rilpivirine hydrochloride	Human immunodeficiency Virus
Abiraterone acetate	Prostate cancer
Avanafil	Erectile dysfunction
Enzalutamide	Prostate cancer
Crofelemer	Patients with diarrhea and HIV infection on antiretroviral therapy
Cobicistat; elvitegravir; emtricitabine; tenofovir disoproxil fumarate	Human immunodeficiency Virus
Radium Ra-223 dichloride	Prostate cancer
Dolutegravir sodium	Human immunodeficiency Virus

^a^As described in detail within the main text

**Table 7:** Indications excluded from selected analyses^a^ on the basis of disproportionately low disease prevalence among nonwhites

Drug	Indication
Ipilimumab	Melanoma
Vemurafenib	Melanoma
Taliglucerase alfa	Gaucher disease
Ivacaftor	Cystic fibrosis
Vismodegib	Basal cell carcinoma
Dabrafenib mesylate	Melanoma
Mipomersen sodium	Homozygous familial hypercholesterolemia
Trametinib dimethyl sulfoxide	Melanoma

^a^As described in detail within the main text

## Abbreviations

FDA: Food and Drug Administration, an agency within the U.S. Department of Health and Human Services that is responsible for protecting the U.S. public health by assuring the safety, effectiveness, quality, and security of human and veterinary drugs, vaccines and other biological products, and medical devices, as well as the safety and security of most of the U.S. food supply, all cosmetics, dietary supplements and products that give off radiation
FDASIA: Food and Drug Administration Safety and Innovation Act, U.S. legislation signed into law on 9 July 2012 that expanded the FDA’s authorities and strengthened the agency's ability to safeguard and advance public health
IQR: interquartile range
ITT: intention-to-treat
n/a: not applicable
WHO: World Health Organization
CI: Confidence Interval
